# Fabrication of self-assembled spherical Gold Particles by pulsed UV Laser Treatment

**DOI:** 10.1038/s41598-018-29661-7

**Published:** 2018-07-26

**Authors:** G. Schmidl, G. Jia, A. Gawlik, J. Kreusch, F. Schmidl, J. Dellith, A. Dathe, Z.-H. Lin, J.-S. Huang, J. Plentz

**Affiliations:** 1 Leibniz Institute of Photonic Technology (IPHT), Albert-Einstein-Straße 9, 07745 Jena, Germany; 2 Friedrich Schiller University of Jena, Institute of Solid State Physics, Helmholtzweg 5, 07743 Jena, Germany; 3 Research Center for Applied Sciences, Academia Sinica, Taipei, 11529 Taiwan; 4 Department of Electrophysics, National Chiao Tung University, Hsinchu, 30010 Taiwan

## Abstract

We report on the fabrication of spherical Au spheres by pulsed laser treatment using a KrF excimer laser (248 nm, 25 ns) under ambient conditions as a fast and high throughput fabrication technique. The presented experiments were realized using initial Au layers of 100 nm thickness deposited on optically transparent and low cost Borofloat glass or single-crystalline SrTiO_3_ substrates, respectively. High (111)-orientation and smoothness (RMS ≈ 1 nm) are the properties of the deposited Au layers before laser treatment. After laser treatment, spheres with size distribution ranging from hundreds of nanometers up to several micrometers were produced. Single-particle scattering spectra with distinct plasmonic resonance peaks are presented to reveal the critical role of optimal irradiation parameters in the process of laser induced particle self-assembly. The variation of irradiation parameters like fluence and number of laser pulses influences the melting, dewetting and solidification process of the Au layers and thus the formation of extremely well shaped spherical particles. The gold layers on Borofloat glass and SrTiO_3_ are found to show a slightly different behavior under laser treatment. We also discuss the effect of substrates.

## Introduction

Nanoparticle (NP) plasmonics have gained importance in recent years in a variety of applications. Plasmonic nanoparticles exhibit Localized Surface Plasmon Resonance (LSPR). LSPR depends on the size, shape and material of the NPs. The applications of LSPR range from enhanced optical spectroscopy, photon management to biophotonic sensing^1–4^. The working principle of nanoparticle plasmonic sensors is typically based on the resonance shift due to the change of the surrounding refractive index or induced particle aggregation and can be used as a sensor principle, for example in investigation of biological binding kinetics^5,6^. Other applications of plasmonic nanostructures include surface enhanced Raman spectroscopy (SERS), optical storage of information, metallic nano-cavities or confinement of electromagnetic fields, as well as broadband solar energy harvesting^7–9^. The manufacturing processes of nanoparticles with different shapes and sizes can be classified into two routes: bottom-up and top-down technologies. They differ, among other things, by their processing complexity. The chemical synthesis as a bottom-up approach is based on growth of nucleation centers whereas organic surfactants stabilize the process. It is preferred for large area processing of bioassays^10–12^, but chemical waste is unavoidable and in most cases, the transfer and arrangement onto substrates lacks in reproducibility. The top-down routes such as electron-beam lithography and photolithography offer the possibility to produce very small, shape defined and exact located particles in the nanometer scale^13,14^, but are disadvantaged with high costs for large area structuring.

An intermediate route, similar to temperature dependent layer dewetting for NP manufacturing^15–17^, uses laser technologies based on energy transfer from laser pulses into a thin metal layer. Utilizing a laser for ablating a solid target or for heating or melting a layer by optical absorption extremely high energy is focused on and will be introduced into the metal. These technologies are used, for instance, to turn spherical nanoparticles into spheroid shape^18^, to prepare alloyed nanoparticles^19,20^ or to produce nanoparticles in liquids or gaseous environment^21–24^ by ablation or melting. An interesting point is the fabrication of alloyed particles. For such process the energy input by short laser pulses, associated with a strong temperature rise, can be a strong advantage. Another advantage of the laser assisted NP fabrication, especially compared to the conventional chemical synthesis of NP, is to generate very pure NPs, since a definite but almost any solid target material with high purity can be used. But, especially when ablated NPs are transferred into an ambient liquid medium by ablation, the agglomeration has to be avoided by, for instance, introduction of organic compounds into the particle solution. This leads to stabilization of the colloid. A surfactant coverage could be overcome by changing the ambient medium to air. A lot of different parameters like the target or layer material with its optical penetration depth, the layer thickness when an deposited continuous layer on a substrate should convert into particles, the ambient medium of the target or of the layer, the wavelength, the laser fluence, the pulse duration and the pulse number affect the particle formation process and thus the results in NP size and size distribution. Such parameters can be used to control the formation process.

Excimer lasers, which emit light in the ultraviolet regime, produce high power pulses with nano-second duration and are ideal for melting thin metallic layers. It has been shown that excimer lasers can produce NP from 5 nm and 10 nm semi continuous metal layers on a silicon wafer^22^. Important processes involved in the NP formation include optical absorption, melting, solidification and ablation of the metal film. A big advantage of using pulsed laser for crystallization is the short melting time, which induces fast raising thermal effects, in contrast to furnace treatment. This is particularly critical for glass substrates, which have a softening point of about 600 °C. Pulsed laser crystallization has been applied, for example, in the manufacturing of polycrystalline solar cells by laser crystallization^25,26^.

The presented work combines UV laser induced fast melting of Au layers on low cost substrates contrary to single crystalline SrTiO_3_ and is a step towards to the fast and cost efficient fabrication of high quality spherical particles. We investigated the influence of laser fluence, laser pulse numbers and substrate materials on the ns-laser assisted particle formation.

## Materials and Methods

### Au film preparation

Using DC magnetron sputtering, Au layers with a thickness of 100 nm were deposited on single-crystalline SrTiO_3_-substrates (STO, a_(STO)_ = 0.3905 nm, thermal conductivity: 12 W/m·K (http://www.azom.com/article.aspx? ArticleID=2362), coefficient of thermal expansion: 9.4·10^−6^/K (http://www.azom.com/article.aspx? ArticleID=2362)) as well as on Borofloat glass substrates (amorphous, thermal conductivity: 1.2 W/m·K (http://www.schott.com), coefficient of thermal expansion: 3.25·10^−6^/K (http://www.schott.com)) without substrate heating. A target-substrate distance (TSD or throw distance) of 95 mm was used in the framework of the present experiments. The argon gas pressure in the deposition chamber was in the range of 15 mTorr with a background pressure of less than 1·10^−6^ Torr. The sputtering power was fixed at 50 W.

### Laser setup for nanoparticle preparation

The main parts of the laser treatment setup consist of a KrF excimer laser, focusing optics, an intensity attenuator, a beam profile homogenizer and an adjustable sample holder. The excimer laser (LPX305, Lambda Physik) emits non-polarized pulses and operates at 248 nm with pulse durations of 25 ns. The pulse-to-pulse repetition rate was set to 1 Hz. The laser induced annealing experiments were carried out with energy densities ranging from 300 mJ/cm^2^ to 1 J/cm^2^, tuned by an intensity attenuator. The number of laser pulses for production of particles was varied between 1 and 10. A laser spot was set to be of about 3.9 × 3.9 mm^2^ with a top head intensity profile created by the focusing optics and the profile homogenizer. The sample position is controlled by an XY translational stage.

### Film and Particle Characterization Methods

For morphological analysis including quantitative analysis of the surface roughness of the initial film, evaluation of the size distribution and the shape of the obtained particles, we have employed atomic force microscopy (AFM, AutoProbe CP AFM system, Park Scientific) and field-emission scanning electron microscopy (FE-SEM, JSM-6300F, JEOL). In order to avoid the charging effect during the SEM investigations it was deposited a 5 nm carbon layer on the substrates, which are covered with particles. During this process the samples were coated at an angle of 60° in a pulsed mode with substrate rotation. The AFM is operated in contact mode with sharpened micro lever tips (tip radius below 10 nm). The analysis of the particle size distribution was carried out with the help of the program ImageJ using SEM images. ImageJ is an open source image processing program designed for scientific multidimensional images. The crystalline structure of the initial Au layers was investigated by X-ray diffraction (XRD, Panalytical X’Pert Pro for crystallite size estimation; Bruker D8 advance for texture analysis) with Cu-Kα_1,2_ (Kα_1_: 1.5406 Å) radiation. The Scherrer equation was applied to estimate the crystallite sizes of the films. For optical characterization of individual nanostructures, a microscope (AxioImager.Z1, Carl-Zeiss) in darkfield-configuration and upright illumination^27,28^ was used with an objective (100x, NA 0.75), where spectroscopy was enabled by coupling a spectrometer (SpectraPro2300i, Princeton Instruments) into the microscopes image plane by an optical fiber, resulting in a circular detection spot of 1 µm in diameter. The raw spectra (*I*_*(raw*)_) are corrected by the background signal next to a particle (*I*_*(bg)*_), the light source (*I*_*(ls)*_) and the system dark current (*I*_*(dc)*_) to obtain the particle scattering spectra (*I*_*(sca)*_) according to the relation *I*_*(sca)*_ = *(I*_*(raw)*_ − *I*_*(bg)*_*)/(I*_*(ls)*_ − *I*_*(dc)*_*)*. The simulations of the spectra and the current distributions were carried out by using the Finite-Difference Time-Domain (FDTD Solutions, Lumerical Solution Inc., Canada) methods.

## Results

Starting point for the experiments are continuous gold layers showing a pronounced reflex of the (111) lattice plane in XRD spectra after deposition independently on the substrate material as it is in general significant for all fcc (face-centered cubic) metals^17,29^. Furthermore, the layers deposited at 15 mTorr and 50 W grow not only highly oriented but also densely packed, resulting in a very smooth surface. The root-mean-square (RMS) noise of the surface roughness measured by AFM is about 1.16 nm, confirming the high flatness of the layers.

**Figure 1:**
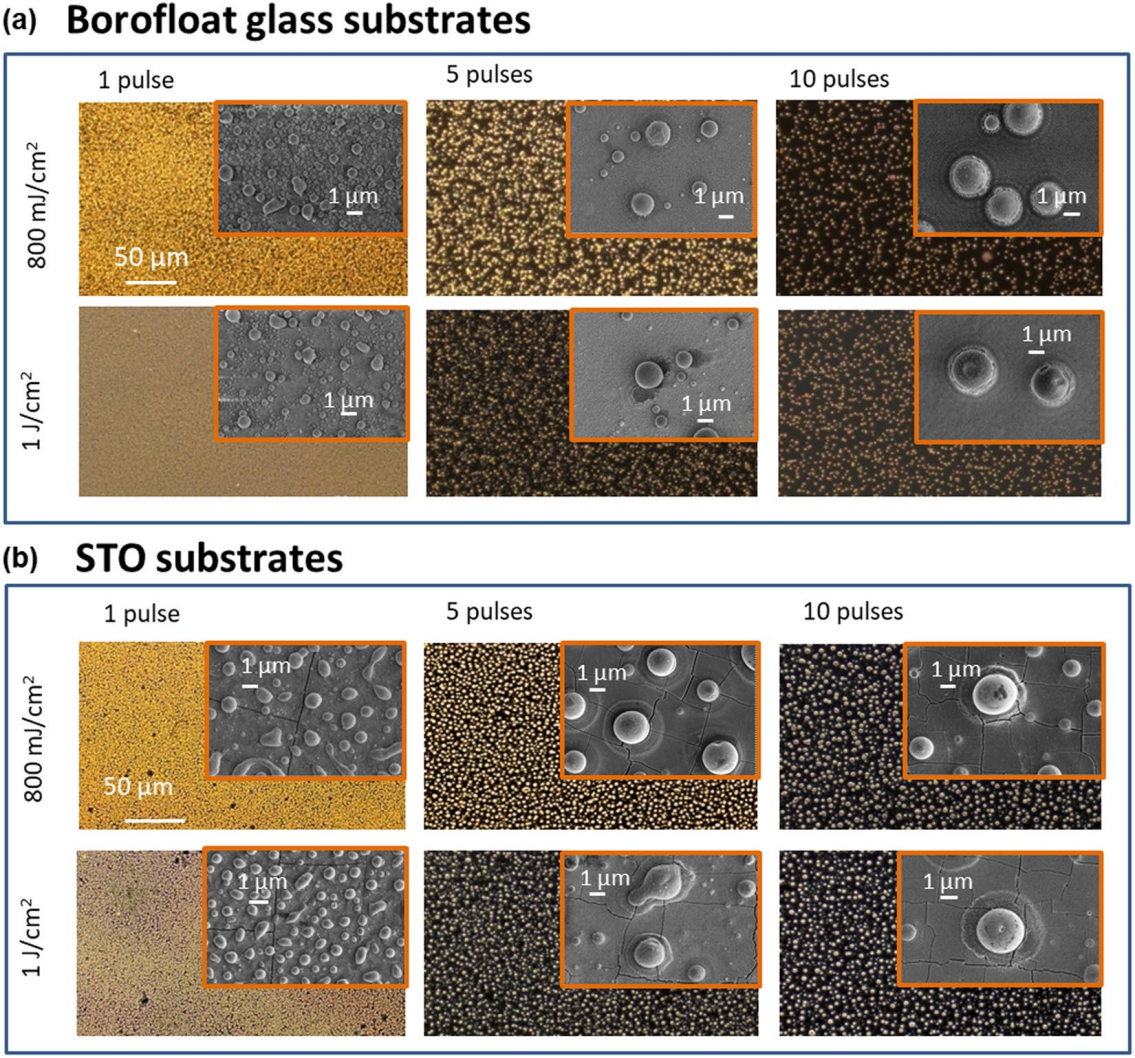
Dependency of particle formation on laser fluence and number of laser pulses. Dark-field microscope images (×50) and the corresponding SEM images (insets: secondary electron image, acceleration voltage = 5 kV) of the laser treated gold layers deposited on Borofloat glass (**a**) and on STO substrates **(b)**. The number of laser pulses is indicated on the top of each column.

The laser induced particle formation process in the UV spectral range is very sensitive to the fluence and pulse number because the absorption of gold in the UV range is more than twice of that in the visible range. This dependency of illumination conditions, together with the effects of the substrate material, determines the morphology of the resulted particles, as visualized in the microscope and SEM images in Fig. 1. It should be additionally mentioned that ambient conditions, the kind of materials and the pulse duration^21^, beside the here described parameters laser fluence, number of pulses or substrate material, are also influencing factors for the production of metal particles by means of lasers. As described in Haustrup *et al*.^21^ fs-pulses can generate smaller particles and narrower particle size distributions than ns-pulses. The results are explained by the far more efficient process of laser-material interaction when using fs-pulses. In that case the electronic system is strongly heated, but an insufficient electron-phonon coupling will be occurred during the time scale of the pulse. That can result in a faster and efficient thermal energy transfer into the lattice.

In general, the time of the energy input and the cooling rate is substantially shorter by using pulsed lasers than for instance in a furnace process. The fast injection of a big amount of energy into the metal films within 25 ns pulse duration – a pulse duration we use in further technology processes - leads to absorption, heating and consequently melting or ablation of the metal from a specific ablation threshold depending on the material. This process is followed by fast solidification of the material after the pulse. This means, the morphology of the resulted particles depends on the laser parameters, metal volume and mainly on the cooling rate. The sphere formation process is basically driven by the minimization of surface energy. At high laser fluence, the process of ablation can occur and is there important for the description of the particle formation.

In a first step we could determine a fluence threshold of *F*_*Th*_ = 500 mJ/cm^2^ – the same for both substrate materials - at which the particle formation process becomes possible. A fluence below this threshold exceeds the melting point of the material in a short time, but it is not enough to convert the gold film into spheres that homogeneously cover the surface. The melted film fuses to create semi-spherical or shapeless particles due to surface tension without a complete surface dewetting. This behavior can be observed in particular with one laser pulse.

**Figure 2:**
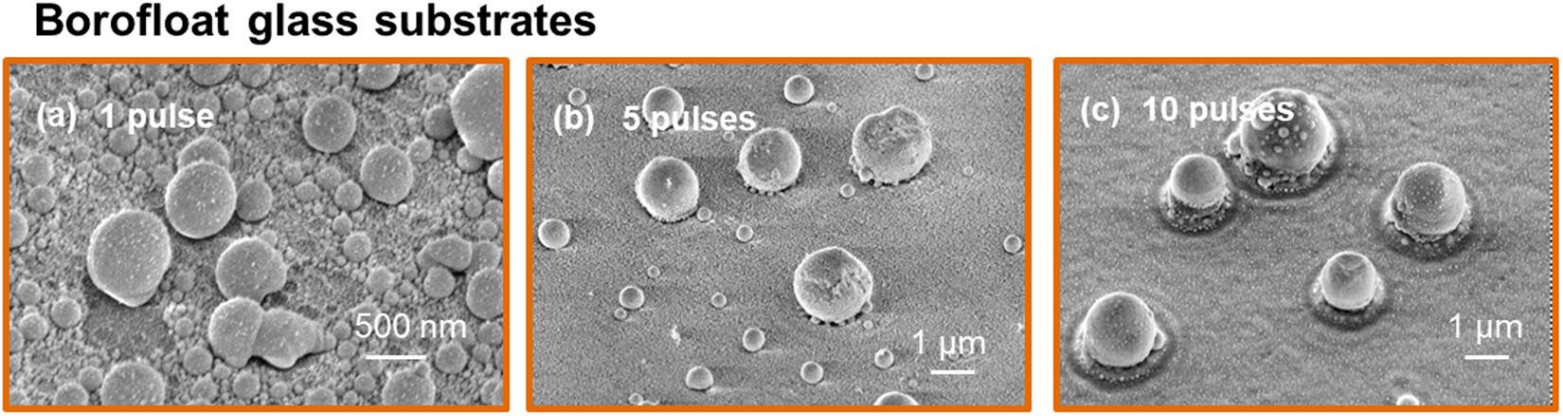
Selected SEM images in a tilted view showing the dependency on the number of pulses. Particles on Borofloat glass, (**a**) 1 laser pulse, (**b**) 5 laser pulses and (**c**) 10 laser pulses are treated by pulsed laser with energy density of 800 mJ/cm^2^. SE images, acceleration voltage = 5 kV, tilt: 45°.

**Figure 3:**
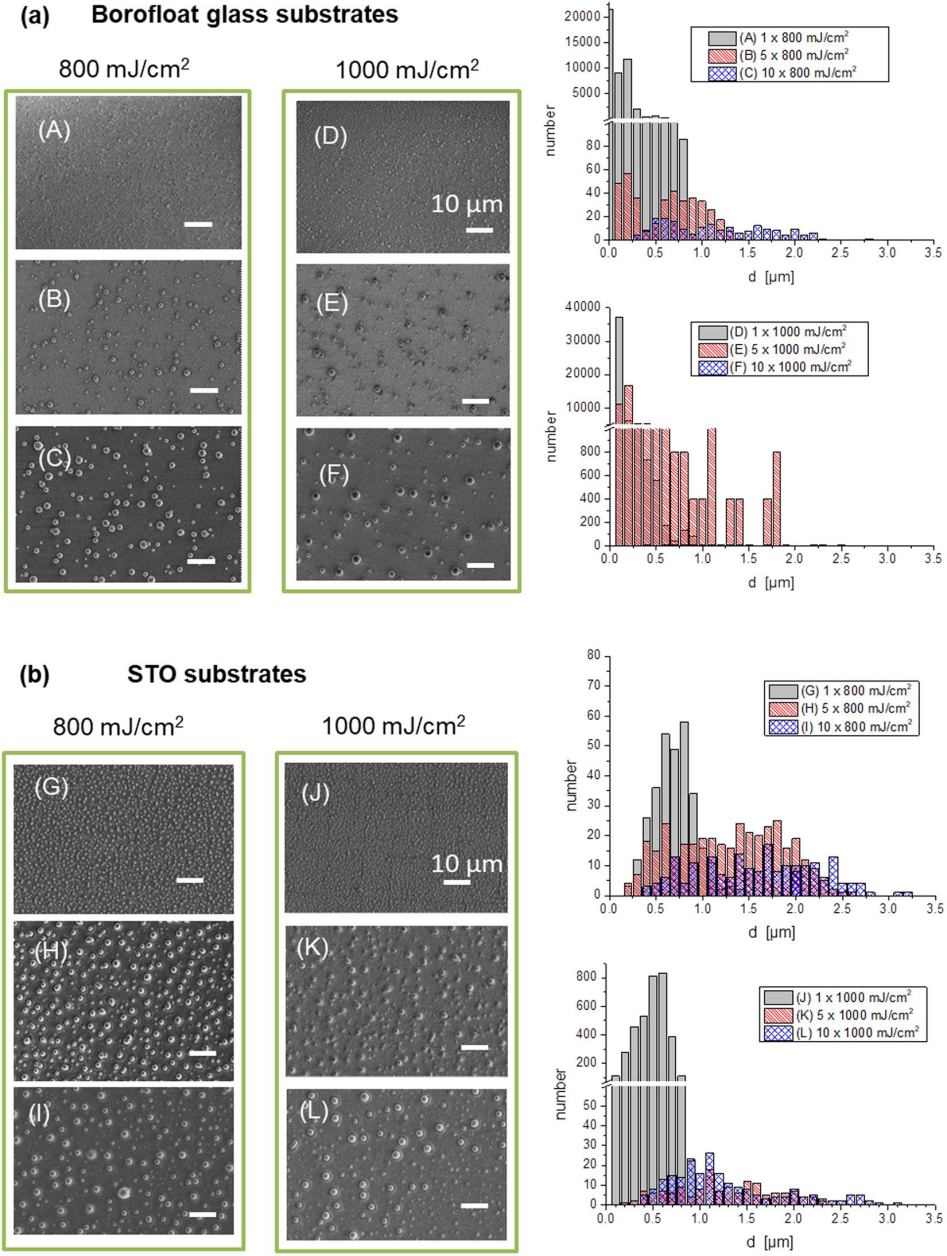
(**a**) SEM images (left: A–F) and corresponding size distributions of particles on Borofloat glass (right) referring to the same area size, (**b**) SEM images (left: G–L) and corresponding size distributions of particles on STO (right) referring to the same area size. Laser fluence: 800 mJ/cm^2^ and 1 J/cm^2^. Laser pulse numbers: 1, 5 and 10. SE images, acceleration voltage = 5 kV. Scale bar of 10 µm is valid for all images.

The next fluence dependent regime observed for F_Th_ > 500 mJ/cm^2^ (Fig. 1) is the window for formation of round spheres with different sizes. Such high fluence can heat the films (first laser pulse) or the already formed particles (subsequent laser pulses) above the evaporation threshold. Ablation can occur and a plume of metal atoms and clusters is observed in the experiment. Clusters can be scattered back to the substrate, because the air serves as a transport barrier. Thus, additional nanoparticles could be formed on the surface or can also adhere on the coarse particles (see Fig. 1(a) top right). In terms of size, shape and size distribution, the process of particle formation is obviously strong dependent on the substrate material in the investigated energy range but also different with regard to the effect of fluence and puls number in these both cases. In Fig. 1 two energy densities above 500 mJ/cm^2^ are presented. In case of glass substrates using one pulse a lot of small particles with a diameter in the range between 200 nm and 500 nm cover the total surface beside some bigger but not spherical ones. By increasing the fluence to 1 J/cm^2^ these still small particles are reduced in size and increased in number. This behavior is partially different in case of STO substrates. Here, the substrate surface is still dewetted using one pulse. An increase in laser energy transferred the particles with a diameter between 500 nm and 1 µm also into smaller particles with diameters ≤500 nm. The effect of an increasing laser fluence above the threshold of 500 mJ/cm^2^ is for both kinds of substrates not recognize so significant than using multiple pulses. As can be seen in the overview in Fig. 1 and in the tilted SEM images in Fig. 2 the number of pulses is also decisive for the surface dewetting and particle formation process. Multiple laser pulses are required to impinge the sample in order to further improve the spherical morphology of particles. The transition from one to five pulses is particularly significant. A single laser pulse is responsible for initializing the dewetting of a continuous metal film and forms only coarse spheres accompanied by a number of smaller particles on the surface in between the bigger ones. Figure 1 shows clearly the difference between particles form on the two substrates, confirming that this process also depends on the substrate material and Fig. 3 summarizes the statistics on the size of particles under various experimental conditions.

As previously noted, while most of the particles with a diameter bigger than 500 nm are formed on the STO substrate even already with one laser pulse (Fig. 3b), very small particles down to the tens of nm range are created on the glass substrate (Fig. 3a). Each subsequent pulse tunes the already formed particles. In general, the number of gold spheres with larger diameters increases with number of laser pulses whereas the surface especially of the glass substrate is still wetted with very small spheres.

A possible explanation for the decrease in number of smaller particles and an increase in number of larger particles, seen clearly in the SEM images and histograms in Fig. 3, is that after fully dewetting further laser pulses supply kinetic energy to the particles, which leads to the migration of particles and merging of small particles to larger ones. The smaller particles move farther and get melted by the laser pulse easier compared to the larger ones.

Moreover, higher number of pulses in general leads to a higher number of spheres with smaller size formed on the coarse particle’s surface. This coverage with small particles was mostly observed on glass substrate and could be attributed to a mass transfer from the repeatedly melted coarse particles to the finer one^21^.

Furthermore, the higher the number of pulses the more pronounced a wave like system around every particle is. Such wave-like structure is shown significantly in the SEM image in Fig. 2(c), where a glass substrate was used. The thermal load on the glass surface may be greater than on STO due to the lower thermal conductivity, so that a surface softening can be presumed (thermal conductivity: STO: 12 W/m·K; Borofloat glass: 1.2 W/m·K). In fact, the surface of STO are found to be smooth and dewetted, in contrast to that of the glass substrate, where the remaining surfaces are covered by several tens of nanometer-sized particles after laser treatment, even after the application of 10 pulses. At the same laser conditions the particle formation process is determined by the surface energies of the substrate materials. When material disappears at high laser energies, this will happens by ablation.

On STO substrates the increase in pulse number leads additionally to an increasingly cracking of the surface. These cracks along the crystal axes can be attributed to the higher thermal expansion of the STO compared to that of glass (coefficient of thermal expansion: STO: 9.4·10^−6^/K; Borofloat glass: 3.25·10^−6^/K). Since high laser energies are used here, it cannot be ruled out that substrate material is also ablated, which could form a shell around spheres.

**Figure 4:**
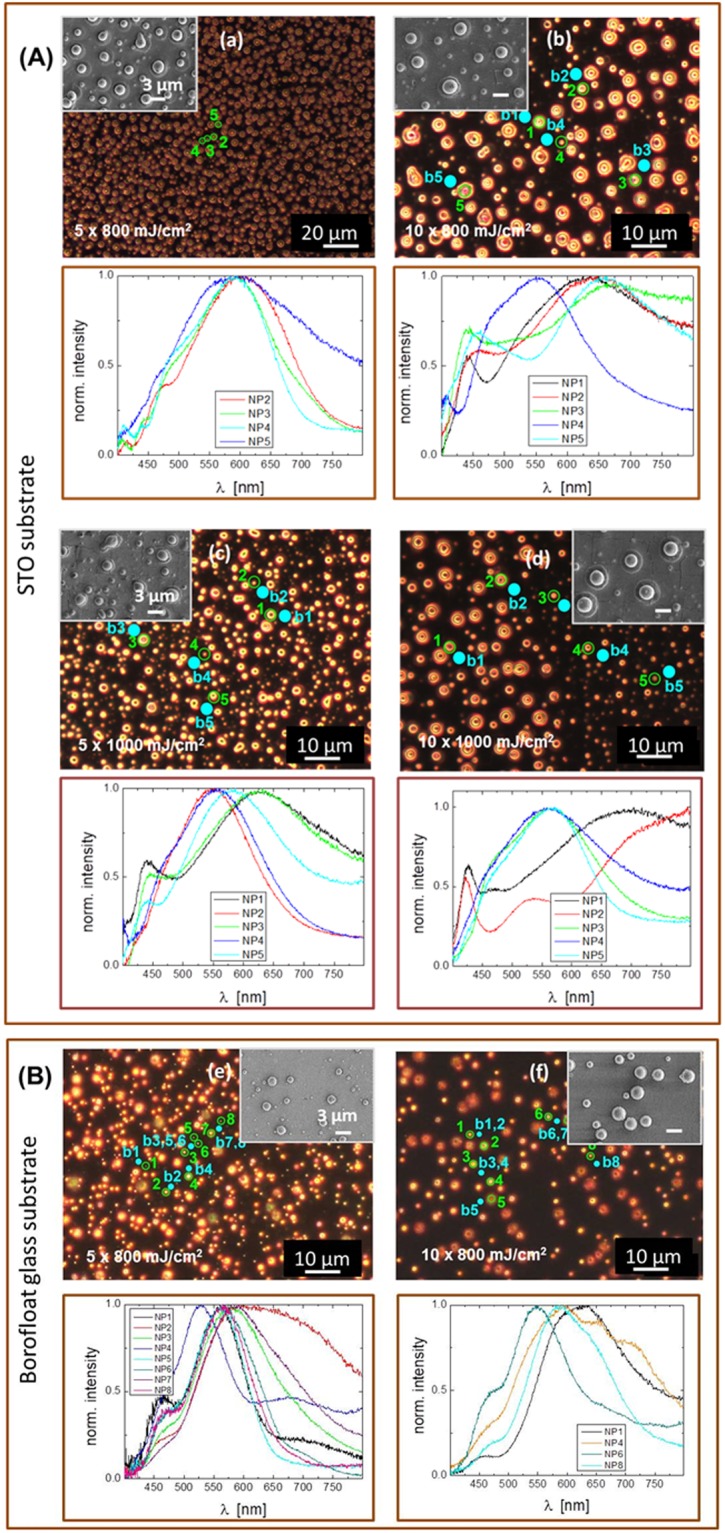
Dark field images and corresponding SEM images of pulsed UV laser induced sphere formation. (**A**) on STO substrates. Laser fluence of 800 mJ/cm^2^ (a,b) and 1000 mJ/cm^2^ (c,d) has been used. (**B**) On glass substrates. Laser fluence of 800 mJ/cm^2^ (e,f) has been used. Laser pulse numbers are 5 and 10 @ 1Hz repetition rate. Numbers (green circles) indicate investigated particles and *b* (blue dots) the location of the corresponding background measurement. Lower panels: Corresponding normalized scattering spectra of single particles.

In the next step, we wanted to show how the laser treatment affects LSPR. Exemplary scattering spectra of single particles with a diameter of some hundred nanometers up to some micrometers as well as the corresponding dark-field images and SEM images of selected surface areas are shown in Fig. 4. For these dark-field scattering experiments non-polarized illumination was used. Localized surface plasmon resonances could be well detected. The spectra partially featured a pronounced additional maximum at ~450 nm. This indicates multipole excitation. The broadening most probably is due to the large particle size but could also arise from overlapping of different LSPR frequencies. Such a hybridization of modes can be caused by adhesion of smaller particles to the big ones or by interaction of particles close to each other, in particular on glass substrates. Furthermore, in contrast to lithography methods the size distribution is broader by self-assembling. Thus the peak positions can differ strongly from particle to particle – especially using glass substrates. But the position can also be influenced by ablation residues from the substrates. These effects could not be clearly separated in this work.

**Figure 5:**
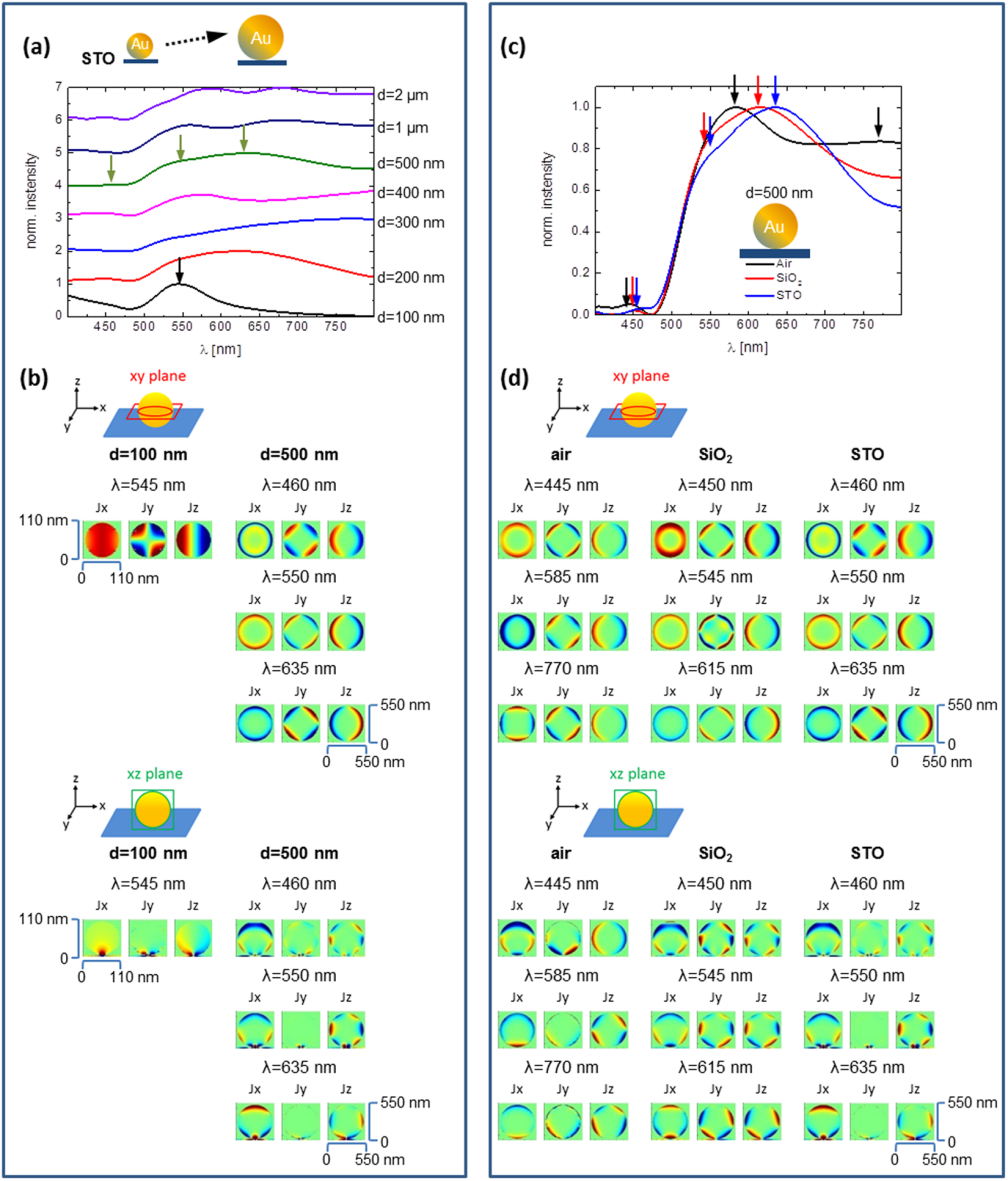
Normalized simulated far-field scattering spectra (**a**,**c**) and specific current density distributions (**b**,**d**) at several resonance frequencies of Au spheres. (**a**,**b**) Comparison between spheres with different size (diameter from 100 nm to 2 μm) on the same STO substrate. (**c**,**d**) Comparison between different substrates (air (n = 1), SiO_2_ (n = 1.47) and STO (n = 2.4)) with the same diameter of 500 nm. To better visualize the current distribution, the images for the sphere with diameter of 100 nm on STO substrate show a square area of 110 × 110 nm^2^ on the xy- and xz-cross sectional planes. All other images of current distribution show an area of 550 × 550 nm^2^.

Since the individual particles prepared under different laser treatment conditions and on different substrates show significant differences in shape, size and spatial arrangement it is important to obtain clear microscope images and SEM images, which provide critical geometrical information to understand their dark-field scattering spectra by numerical simulations. For interpretation of the complex spectral behavior, we have carried out simulations using finite-difference time-domain (FDTD Solutions, Lumerical Solution Inc., Canada) method to undertstand the scattering peaks. The FDTD calculations shown in Fig. 5 were solved for the particles far-field response as well as for the current density distributions (Jx, Jy, Jz) inside the gold spheres recorded at the xy-plane and xz-plane cutting through the center of the sphere. The simulations allow us to understand the resonance and the changes in the spectra caused by size and substrate. In Fig. 5(a) the influence of the sphere size on top of a STO substrate is presented. The particle size used for simulation is between 100 nm and 2 µm. Representative diameters are used according to the actual size distribution of the particles produced with our laser treatment method.

There are three major size effects. First, increasing the size shifts the resonance to longer wavelength. For example, the fundamental dipole-mode of a 100 nm sphere is at around 545 nm. This mode shifts to 630 nm when the diameter increases to 200 nm (red trace in Fig. 5(a)). Secondly, higher order modes emerge as the size of the sphere becomes significantly larger than the effective wavelength of light. With increasing particle diameter higher modes are getting more pronounced in the spectra. This is visible in the formation of additional resonances at different wavelengths.

As can be seen in Fig. 5(b), for a 100 nm sphere, the dipole resonance is dominant, while multiple peaks start to show up as the diameter of the sphere increases to 400 nm. These peaks correspond to higher order modes^30–32^. For spheres with diameter larger than 500 nm, the mode order is very high and the current distribution becomes very complicated. The modes cannot be easily assigned by considering the current distribution only in xy -plane or xz-plane cutting the sphere. Here, we show both the distribution of three current components in xy- and xz- plane cutting through the center of the sphere to illustrate the resonance of different modes. Although some modes show similar current distributions in xy-plane, the current distributions in xz-plane are not the same. These modes are higher order spherical harmonic modes of surface plasmon on large gold spheres. Three-dimensional current distributions are needed to fully understand the modes, which is beyond the scope of this work. Thirdly, for large gold spheres with a radius larger than the penetration depth of light, the gold sphere becomes more like a spherical shell resonator because the currents can only be induced within a shell of finite thickness, as can be seen from the current distribution in Fig. 5(b). This makes the analysis of the mode even more complex.

For the substrate effect, a gold sphere (diameter = 500 nm) has been put onto three different substrates, namely air (n = 1), SiO_2_ (n = 1.47) and STO (n = 2.4). The simulated scattering spectra are summarized in Fig. 5(c) and the current distributions in xy- and xz-plane are shown in Fig. 5(d). The dependency of the peak position on the surrounding medium indicates that the higher the refractive index, the more the dipole mode is red shifted. For example, the major peak of the sphere in air around 585 nm red-shifts to 615 nm for SiO_2_ substrate and to 635 nm for STO substrate. This red-shift is due to the fact that the effective wavelength of the excitation light reduces as the index of surrounding medium increases, leading to the increase of the effective sphere size. In the case of glass substrates, the emerging of additional resonance peaks might be due to the influence of closely spaced particles. The experimental spectra are more complex than the simulated ones because the experimental spectra are not obtained from single particles. In principle, a coupling of more than two particles, for instance of “piggyback particles”, can also be responsible for a spectral broadening and observation of additional resonances by formation of hybridized modes. More detailed simulations and modelling are needed to understand the spectrum of large gold spheres, which are however beyond the scope of this work about laser treatment fabrication.

## Conclusions

It has been demonstrated that starting from a 100 nm thick gold layer spherical Au particles from smaller than hundreds of nanometers to a few micrometers can be produced on glass as well as on SrTiO_3_ substrates. The fabricated single spheres, which were formed during a self-assembling process show LSPR spectra dependent on the particle size. The homogeneity of the particle size distribution and thus the reproducibility of the LSPR spectra of the individual particles can be influenced by the treatment conditions, including the laser fluence and number of pulses. The described fast fabrication process based on an excimer laser treatment is connected with a rapid induce of a big amount of heat into metal films leading to metal melting up to an ablation threshold dependent on the laser fluence and with a fast solidification. A limit of 500 mJ/cm^2^ was determined to initialize a particle formation. We also found that multiple laser pulses are needed to form well shaped gold spheres. In contrast to STO substrates, the laser treatment of a gold layer on glass substrates is accompanied with the generation of nm-sized nanoparticles in between the bigger ones. This method can be automatized by placing the film on a positioning stage to assemble the irradiated areas and to create a large particle-coated area. An interesting point for using short laser pulses is the fabrication of alloyed particles. For such process the short and high energy input associated with a strong temperature rise and a short solidification phase can be a strong advantage. In this publication we focused mainly on tuning the laser conditions to obtain optimal particle formation. The possible impact of the original film thickness is indeed another factor influencing the process of laser assisted particle fabrication. The volume of material can be important with respect to the heating and, thus, to the melting and solidification process. It can influence the dewetting behavior and can result in different sizes of nanoparticles. Moreover, thin discontinuous films (islands) can introduce more random fluctuation compared to a continuous films and influence so the optimal laser conditions.

## References

[1] Li M, Cushing SK, Wu N (2015). Plasmon-enhanced optical sensors: A review. Analyst..

[2] Xu Y, Xuan Y, Yang L (2015). Full-spectrum photon management of solar cell structures for photovoltaic–thermoelectric hybrid systems. Energy Conversion and Management.

[3] Zhao J, Zhang X, Yonzon CR, Haes AJ, Van Duyne RP (2006). Localized surface plasmon resonance biosensors. Nanomedicine.

[4] Hall WP, Ngatia SN, Van Duyne RP (2011). LSPR biosensor signal enhancement using nanoparticle−antibody conjugates. J. Phys. Chem. C.

[5] Jain PK, Huang X, El-Sayed IH, El-Sayed MA (2007). Review of some interesting surface plasmon resonance-enhanced properties of noble metal nanoparticles and their applications to biosystems. Plasmonics.

[6] Petryayeva E, Krull UJ (2011). Localized surface plasmon resonance: Nanostructures, bioassays and biosensing - A review. Anal. Chim. Acta.

[7] Mulvihill MJ, Ling XY, Henzie J, Yang P (2010). Anisotropic etching of silver nanoparticles for plasmonic structures capable of single-particle SERS. J. Am. Chem. Soc..

[8] Siozios A (2012). Optical encoding by plasmon-based patterning: hard and inorganic materials become photosensitive. Nano Lett..

[9] Atwater HA, Polman A (2010). Plasmonics for improved photovoltaic devices. Nat. Mater..

[10] Zhao P, Li N, Astruc D (2013). State of the art in gold nanoparticle synthesis. Coord. Chem. Rev..

[11] Aherne D, Ledwith DM, Gara M, Kelly JM (2008). Optical properties and growth aspects of silver nanoprisms produced by a highly reproducible and rapid synthesis at room temperature. Adv. Funct. Mater..

[12] Sun Y, Xia Y (2002). Shape-controlled synthesis of gold and silver nanoparticles. Science.

[13] Barbillon G (2007). Electron beam lithography designed chemical nanosensors based on localized surface plasmon resonance. Surf. Sci..

[14] Hicks EM (2005). Controlling plasmon line shapes through diffractive coupling in linear arrays of cylindrical nanoparticles fabricated by electron beam lithography. Nano Lett..

[15] Bernhardt H (2015). Engineering crystalline Au nanoparticles of anisotropic shape in epitaxially grown high-index SrTiO_3_. J. Mater. Sci..

[16] Schmidl G (2015). Formation and characterization of silver nanoparticles embedded in optical transparent materials for plasmonic sensor surfaces. Mater. Sci. Eng. B.

[17] Kracker M, Wisniewski W, Rüssel C (2014). Texture of Au, Pt and Pd/PdO nanoparticles thermally dewetted from metal layers on fused silica. RSC Adv..

[18] Hubenthal F (2009). Tailor-made metal nanoparticles as SERS substrates. Appl. Phys. B.

[19] Hodak JH, Henglein A, Giersig M, Hartland GV (2000). Laser-Induced Inter-Diffusion in AuAg Core−Shell Nanoparticles. J. Phys. Chem. B.

[20] Yao Y (2018). Carbonthermal shock synthesis of high-entropy-alloy nanoparticles. Science.

[21] Haustrup N, O’Connor GM (2011). Nanoparticle generation during laser ablation and laser-induced liquefaction. Phys. Proc..

[22] Kalgagiannis N (2016). Selective modification of nanoparticle arrays by laser-induced self-assembly (MONA-LISA): putting control into bottom-up plasmonic nanostructuring. Nanoscale.

[23] Hahn A, Barcikowski S, Chichkov BN (2008). Influences on nanoparticle production during pulsed laser ablation. J. Laser Micro Nanoen..

[24] Kim, M., Osone, S., Kim, T., Higashi, H. & Seto, T. Synthesis of nanoparticles by laser ablation: A review. *Kona Powder Part. J*, 10.14356/kona.2017009 (2016).

[25] Plentz J (2014). Polycrystalline silicon thin-film solar cells prepared by layered laser crystallization with 540 mV open circuit voltage. Thin Solid Films.

[26] Michaud JF (2016). Laser irradiation influence on Si/3C-SiC/Si heterostructures for subsequent 3C-SiC membrane elaboration. MRS Advances (Electronics and Photonics).

[27] Siedentopf H, Zsigmondy R (1902). Über Sichtbarmachung und Größenbestimmung ultramikoskopischer Teilchen, mit besonderer Anwendung auf Goldrubingläser. Ann. Phys..

[28] Katzer C (2012). YBa_2_Cu_3_O_7-δ_ matrix-induced *in situ* growth of plasmonic Au nanoparticles for biological sensor devices. J. Nanopart. Res..

[29] Schmidl G (2017). Confocal sputtering of (111) orientated smooth gold films for surface plasmon resonance approaches. Vacuum.

[30] Ruan Q, Shao L, Shu Y, Wang J, Wu H (2014). Growth of Monodisperse Gold Nanospheres with Diameters from 20 nm to 220 nm and Their Core/Satellite Nanostructures. Adv. Opt. Mater..

[31] Rodriguez-Fernández J, Pérez-Juste J, Javier García de Abajo F, Liz-Marzán LM (2006). Seeded Growth of Submicron Au Colloids with Quadrupole Plasmon Resonance Modes. Langmuir.

[32] Liang C-C (2011). Plasmonic metallic nanostructures by direct nanoimprinting of gold nanoparticles. Opt. Express.

